# Fine particulate matter (PM_2.5_) and suicide in South Korea: an instrumental variable analysis using thermal inversion

**DOI:** 10.3389/fpubh.2026.1909365

**Published:** 2026-09-14

**Authors:** Osung Kwon

**Affiliations:** Department of Resource Economics, University of Massachusetts Amherst, Amherst, MA, United States

**Keywords:** fine particulate matter (PM2.5), health, instrumental variable analysis (IVA), suicide mortality, thermal inversion

## Abstract

Air pollution causes the deterioration of both physical and mental health and increases the risk of suicide. Existing studies on the association between air pollution and suicide rely on meta-analyses and simple correlation analyses, which cannot quantify how much the increase in air pollution levels increases suicide rates. This study thus examines the relationship between fine particulate matter (PM_2.5_) pollution and suicide mortality in South Korea from 2015 to 2019 using thermal inversions as an instrumental variable to induce exogenous variations in PM_2.5_ levels. The findings suggest that a 1 μg/m^3^ increase in PM_2.5_ pollution is associated with a rise of 0.6628 suicide deaths per 1 million population (*β* = 0.6628, 95% CI: 0.1049–1.2208). The 2.94% increase in suicide mortality highlights the significant health impact of PM_2.5_ exposure. Additionally, this study shows the differentiated effects of exposure to PM_2.5_ on the suicide mortality rate. An increase in PM_2.5_ was associated with higher suicide mortality (deaths per million) in males (7.873), older adults (21.18), individuals with lower education levels (4.091), and those living in non-metropolitan areas (5.434). These impacts are observed among several groups: older adults, males, adults without a college education, and people living in non-metropolitan areas. These results provide empirical evidence for the heterogeneous effect of PM_2.5_ pollution on the suicide mortality rates across demographic groups.

## 1 Introduction

Understanding how air pollution affects public health is essential from both the individual and social perspectives. From an individual perspective, pollution affects both physical and mental health, causing people to suffer from physical diseases such as respiratory and cardiovascular diseases (1, 2), and is associated with worsening depressive symptoms, increased stress levels, and reduced cognitive abilities (3, 4). Evidence also indicates that pollution exposure can adversely affect psychological well-being and elevate suicide risk (5, 6). South Korea has the highest suicide rate among OECD countries, with suicide being a major cause of death among people in their teens, twenties, and thirties (7). Recognizing suicide as a serious social problem, the South Korean government established the National Suicide Prevention Plan as a significant health policy task to reduce the suicide rate (8). In this context, empirically examining the relationship between air pollution and suicide is important to protect individual health and social security.

This study analyzes the effect of air pollution on the suicide rate in South Korea using district-level monthly data from 2015 to 2019, focusing on fine particulate matter (PM_2.5_), which penetrates the human respiratory system.^1^ Specifically, I adopted an instrumental variable (IV) approach using thermal inversions as an IV to address endogeneity in air pollution. Thermal inversion occurs when a layer of hot air overlies cold air, trapping air pollutants originating from the surface. This meteorological phenomenon significantly affects air pollution and is independent of economic activity (3). Several studies have used inversion as an instrumental variable to isolate the effects of air pollution (9–12).

The first-stage regression showed that thermal inversion was positively associated with pollution levels, with each additional event of thermal inversion per month associated with a 0.0446 increase in PM_2.5_ (95% CI: 0.0267–0.0625). The Wald F-statistic was greater than the rule of thumb, demonstrating the instrument’s robustness and relevance. The second stage regression estimated that a 1 microgram per cubic meter (μg/m^3^) increase in PM_2.5_ pollution is associated with 0.6628 (95% CI: 0.1049–1.2208) additional deaths by suicide per 1 million population in a month, corresponding to a 2.94% increase relative to the average monthly suicide rate. An increase in PM_2.5_ by one standard deviation (8.25 μg/m^3^) is estimated to be 5.465 additional deaths per million people. The IV estimates were significantly larger than the ordinary least squares (OLS) estimates, suggesting potential bias in studies related to pollution exposure (1, 13).

This study conducted a heterogeneity analysis accounting for demographic characteristics associated with the effects of pollution on suicide rates. Based on an analysis by gender and age group, a 1-unit increase in PM_2.5_ increased the male suicide rate by 0.9549 (95% CI: 0.1472–1.7627); the estimate for older adults was positive (2.5685) but statistically significant only at the 10% level, with a confidence interval including zero (95% CI: −0.1392–5.2763). Additionally, the analysis by education level and metropolitan area status showed that a 1 μg/m^3^ rise in PM_2.5_ led to an increase of 0.4962 (95% CI: 0.0525–0.9399) in the suicide rate for individuals who have a high school education or lower and an increase of 0.6590 (95% CI: 0.0289–1.2891) among those in non-metropolitan areas. These results are consistent with those of previous studies. For instance, Persico and Marcotte (6) show that the effects of pollution on suicide mortality are significant for white persons, males, older individuals, and those living in rural areas. Molitor et al. (5) prove that PM_2.5_ exposure in the US more strongly affects the suicide rate among those in rural counties.

This study found differential effects of PM_2.5_ across demographic factors among men, working-age adults, and individuals without a college education. As such, it makes several key contributions to the literature based on its theoretical background and empirical analysis. First, it offers relevant social and policy implications, owing to its focus on South Korea, which registers a high suicide rate. Specifically, this is the first empirical analysis using 239 districts in South Korea to provide evidence on the relationship between PM_2.5_ concentration and suicide rate. Lee et al. (14) analyze the relationship between air pollutants and suicide rates in 23 South Korean cities by excluding PM_2.5_ concentration, and find limitations in representing South Korea’s suicide rate based on a restricted number of cities and in the absence of PM_2.5_, the pollutant most directly associated with health outcomes. Other studies in South Korea have found a positive correlation between air pollution, suicide (14–16), and mental health indicators (17, 18). However, extant studies have limitations in terms of interpreting causal relationships and providing evidence of actual suicide mortality. As such, this study provides evidence consistent with a potential mechanism for the effect of PM_2.5_ pollution on the suicide rate using thermal inversions based on nationwide district-level data with broad national geographic coverage.

Second, this study analyzes the heterogeneous impact of PM_2.5_ pollution on suicide mortality based on relevant demographic factors reflecting South Korea’s social situation of transitioning into an aging society, with a changing family structure and a population increasingly concentrated in metropolitan areas. These significant social changes also cause mental health to deteriorate and increase the risk of suicide. Lee et al. (19) report that changing the family structure increases older-adult suicide owing to social isolation, while Hwang et al. (20) observe that old age and lower education contributed to increasing suicide from 2015 to 2020 in South Korea. The findings of this study find statistically significant associations between PM_2.5_ pollution and suicide mortality among males, older adults, individuals with lower education, and those in non-metropolitan areas and environmental health policies.

Third, this study contributes to the literature on suicide by indicating that PM_2.5_ pollution is an important factor. Previous studies have found a positive relationship between air pollutants and suicide using case-crossover analyses or meta-analyses. Case-crossover studies have found short-term associations (21–24), and meta-analyses have synthesized prior findings (25, 26). However, these approaches are constrained by confounding factors and methodological limitations in explaining causality. As such, this study uses thermal inversion, which prevents vertical air circulation, as an exogenous shock that increases PM_2.5_ pollution. While Persico and Marcotte (6) show that a 1 μg/m^3^ increase in daily PM_2.5_ is associated with a 0.49% increase in daily suicides in the US between 2003 and 2010 and Molitor et al. (5) investigate how increased air pollution leads to an increase in suicides in rural areas, finding that a 1 μg/m^3^ rise in PM_2.5_ caused by wildfire causes 0.27 additional suicide deaths per million inhabitants, this study provides additional evidence that PM_2.5_ is associated with suicide mortality in South Korea.

## 2 Background

### 2.1 Suicide and air pollution

Suicide is a significant global public health problem and is influenced by various factors, including mental health, socioeconomic environment, and stressors. Air pollution has recently attracted increasing attention as a relevant factor, with studies reporting that it can harm the human nervous system and mental health beyond its effects on respiratory and cardiovascular diseases (3). Indeed, it can cause various psychological problems such as depression, anxiety disorders, and stress responses, thus potentially increasing the risk of suicide.

Ambient air pollutants are an important contributing factor to suicide. Lian et al. (21) report that short-term exposure to air pollution (NO_2_, SO_2_, and PM_2.5_) is associated with increases in excess suicide mortality of 8.07, 8.07, and 1.55%, respectively. Davoudi et al. (25) explain that the average 10 μg/m^3^ increases in air pollutants (NO_2_, SO_2_, PM_10_, and PM_2.5_) is associated with increased suicide rates on the same day and the previous day. Yang et al. (24) find that an interquartile range increase in O_3_ pollution corresponds to a 11% increase in suicide risk on warm days and a 15% increase on cool days. This indicates that exposure to O_3_ contributes to daily variations in suicide. Air pollutants, including PM_2.5_, PM_10_, and NO_2_, are positively correlated with suicide mortality, and their effects are more pronounced at higher temperatures (23, 26).

South Korea has the fourth-highest suicide rate in the world and the highest rate among OECD countries (27). The leading cause of suicide in South Korea is mental problems (39%), followed by economic difficulties (24.2%) and physical illness (17.7%) (7). In 2023, the suicide rate for males was 3.9 times higher than that for females, being the largest among the older-adult population (28). Kim et al. (29) reported that, in South Korea in 2013, suicide rates were higher among males than females, and the risk of suicide increased with age. Shin et al. (30) examined 1,548 individuals aged over 60 years and found that suicide is strongly associated with depression. These findings suggest that South Korea is a pertinent case study for suicide research and highlight the need for an approach that analyzes demographic factors to understand the country’s high suicide rate. Figure 1 plots the monthly average suicide rate in South Korea from 2015 to 2019. The suicide rate has gradually increased from 20 per million in 2015 to 25 per million in 2019. This rate is not only subject to short-term variations, but also reflects a long-term increase, emphasizing its significance as a pressing public health concern.

**Figure 1:**
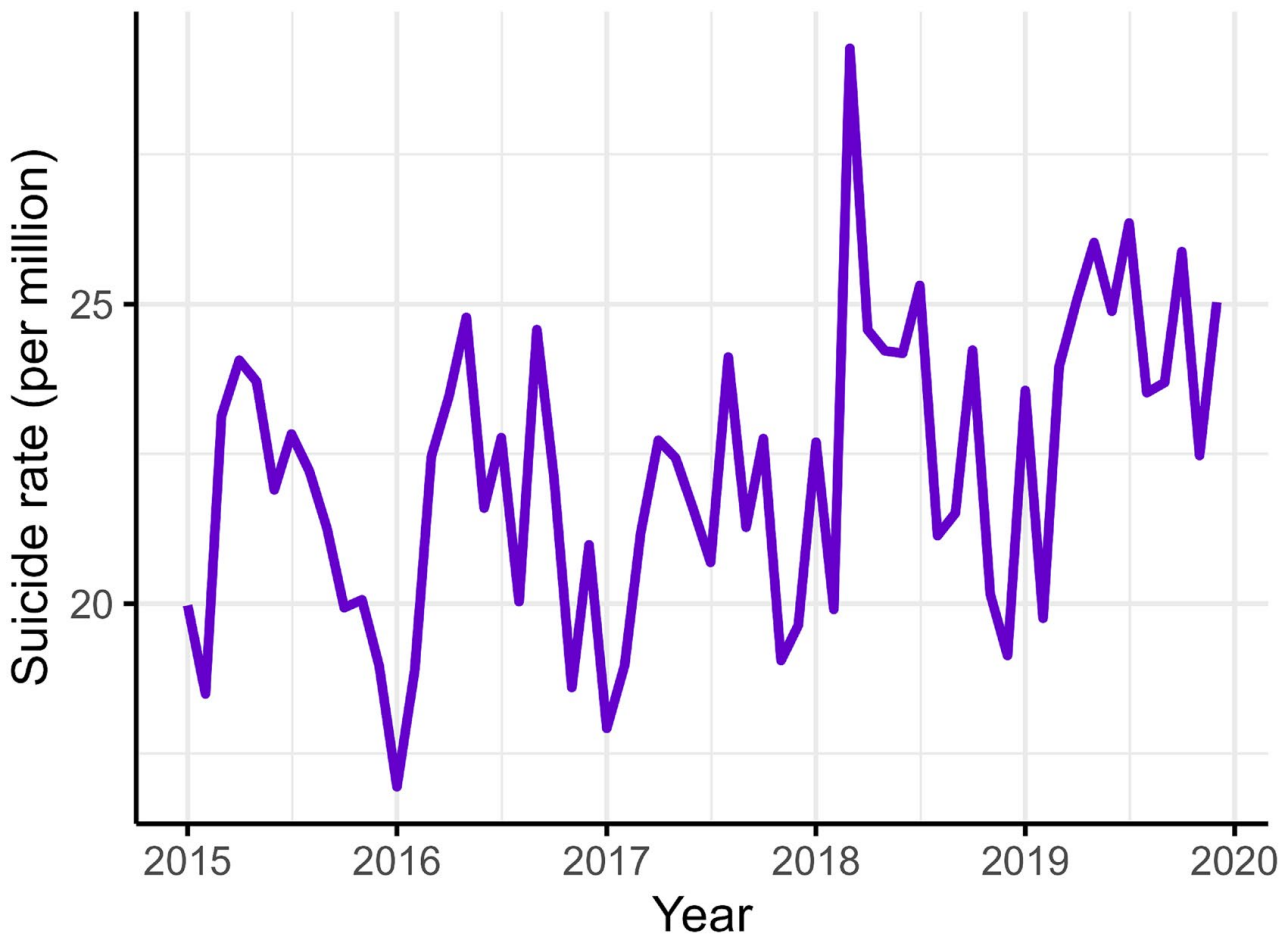
Time trend of the suicide rate in South Korea (2015–2019). This figure presents the district-level average suicide rates in South Korea between 2015 and 2019. The suicide rate is defined as the number of suicide deaths per 1 million people.

Three direct channels through which air pollution increases suicide risk have been proposed (6). The mechanisms through which air pollution may elevate suicide risk can be understood in terms of physiological, cognitive, and behavioral pathways. First, exposure to PM_2.5_ induces inflammatory responses and oxidative stress, which affect the central nervous system and consequently heighten depression, anxiety, and stress (31, 32). These physiological changes may undermine mental health and, in turn, increase vulnerability to suicide. Second, air pollution may impair cognitive functioning and decision-making ability. Previous studies have shown that PM_2.5_ exposure diminishes cognitive performance and amplifies impulsivity (33, 34), thereby increasing the likelihood of extreme outcomes, such as suicide. Third, air pollution may also alter individual behavior. During periods of elevated particulate matter, outdoor activities as well as social interactions decline, which can intensify social isolation and adversely affect mental health. These effects are especially pronounced among populations that engage heavily in outdoor activities or among socially vulnerable groups (5, 6). Collectively, these physiological, cognitive, and behavioral pathways provide a theoretical basis for understanding how PM_2.5_ exposure may shape suicide risk, thereby offering a structured account of the empirical associations documented in prior studies.

Air pollution levels tend to fluctuate simultaneously with economic activity, weather conditions, and seasonal factors, all of which affect suicide rates. For example, economic recession can increase suicide rates and reduce air pollution, reflecting the possibility of reverse causality. In addition, measurement error in air pollution data can also represent a bias factor. Therefore, an IV approach using exogenous variations is useful for examining the relationship between air pollution and suicide rates.

### 2.2 Thermal inversion

Thermal inversion is a common atmospheric phenomenon observed under ordinary weather conditions. Although it is common for air temperature to decrease with increasing altitude under normal atmospheric conditions, a thermal inversion occurs when the general air temperature gradient is reversed, resulting in a warm air layer settling on top of the cold air layer. This phenomenon can occur for various reasons, such as radiation, subsidence, and advection inversions (11). Figure 2 compares the normal atmospheric pattern and thermal inversion, explaining how warm air layers prevent vertical dispersion. The vertical circulation of air is restricted when a thermal inversion layer forms, preventing air pollutants emitted from the surface from spreading into the upper layers. This means that they remain in a layer close to the surface, deteriorating air quality. Fu et al. (10) estimate that the annual number of days with thermal inversions increases the annual average PM_2.5_ levels by 5.3%, while Arceo et al. (9) find that each additional thermal inversion is associated with an increase in weekly average PM_10_ and carbon monoxide (CO) concentrations of 5.7 and 6.3%, respectively. Chen et al. (3) show that one additional thermal inversion during the past month increases PM_2.5_ concentrations by 0.30 μg/m^3^, or 0.63%, evaluated at the mean. These findings underline the role of thermal inversions as a meteorological factor of air pollution, and highlight that understanding the frequency or intensity of thermal inversions is essential for examining variations in air quality and their potential health impacts.

**Figure 2:**
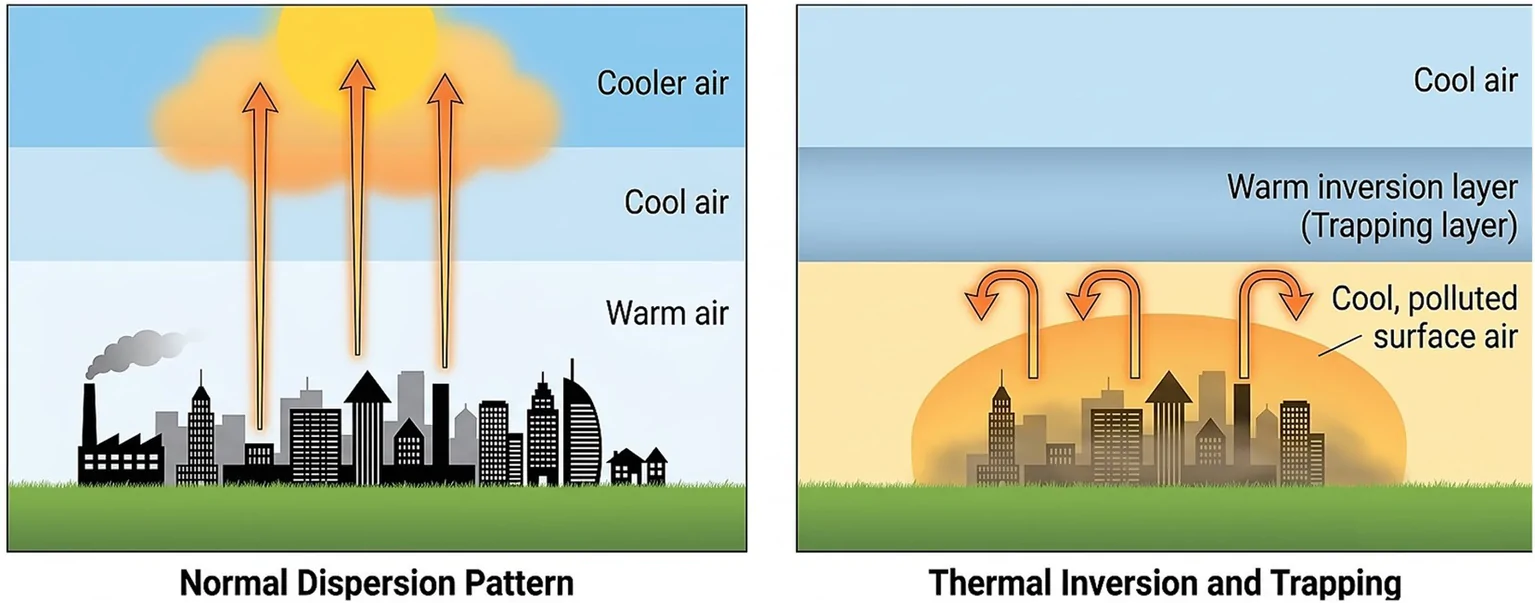
Normal atmospheric pattern vs. thermal inversion.

This study utilizes thermal inversions as an IV to help address endogeneity in air pollution. Existing studies leverage thermal inversions as plausibly exogenous variations in pollution levels. For instance, Jans et al. (12) use inversion episodes causing pollution changes to study the impact of PM_10_ on children’s respiratory health problems, and Sager (35) utilizes temperature inversions to examine the effect of PM_2.5_ on road traffic accidents in the UK. Arceo et al. (9) use an instrument to measure PM_10_ and CO pollution levels and thermal inversions to estimate their impact on infant mortality in Mexico City. These studies support the use of thermal inversions as a relevant instrument for isolating the exogenous variations in pollution levels. Based on this mechanism, this study leveraged thermal inversions to help address potential endogeneity in the relationship between air pollution and suicide mortality in South Korea.

## 3 Materials and methods

### 3.1 Data

This study used monthly data on suicide mortality, air pollution, and weather variables from 2015 to 2019 from multiple sources across all districts of South Korea. Air pollution is measured using monthly averages, and weather variables are constructed by aggregating daily observations into monthly measures.

#### 3.1.1 Suicide mortality data

Cause-of-death data collected from all death certificates and resident registration data between 2015 and 2019 were used to calculate the suicide mortality in each district of South Korea. These data contain individual information, such as the date of death, gender, age group, address, education level, and cause of death. Deaths from all local government offices were aggregated at the district-by-month level based on the Korean Standard Classification of Diseases and Causes of Death (KCD) from Statistics Korea. The number of deaths was divided by the district-level population provided by resident registration records to calculate suicide mortality. The measure of suicide mortality, defined as the number of suicide deaths per 1 million people, was generated using the KCD code to indicate intentional self-harm (suicide).^2^ Although suicide counts are discrete, the district-level mortality rate is zero in only 6.6% of district-month observations for the total population. At this level of aggregation, it is well approximated by a continuous measure, so a linear specification is appropriate for the main analysis. Sparsity is more pronounced for the youngest age group, as discussed in the Results section.

Figure 3 shows the average district-level suicide rates over the study period. Darker shades of purple indicate higher suicide rates, suggesting the heterogeneity of suicide rates across districts. This indicates that South Korea’s suicide rate in suburban areas is higher than that in urban areas.

**Figure 3:**
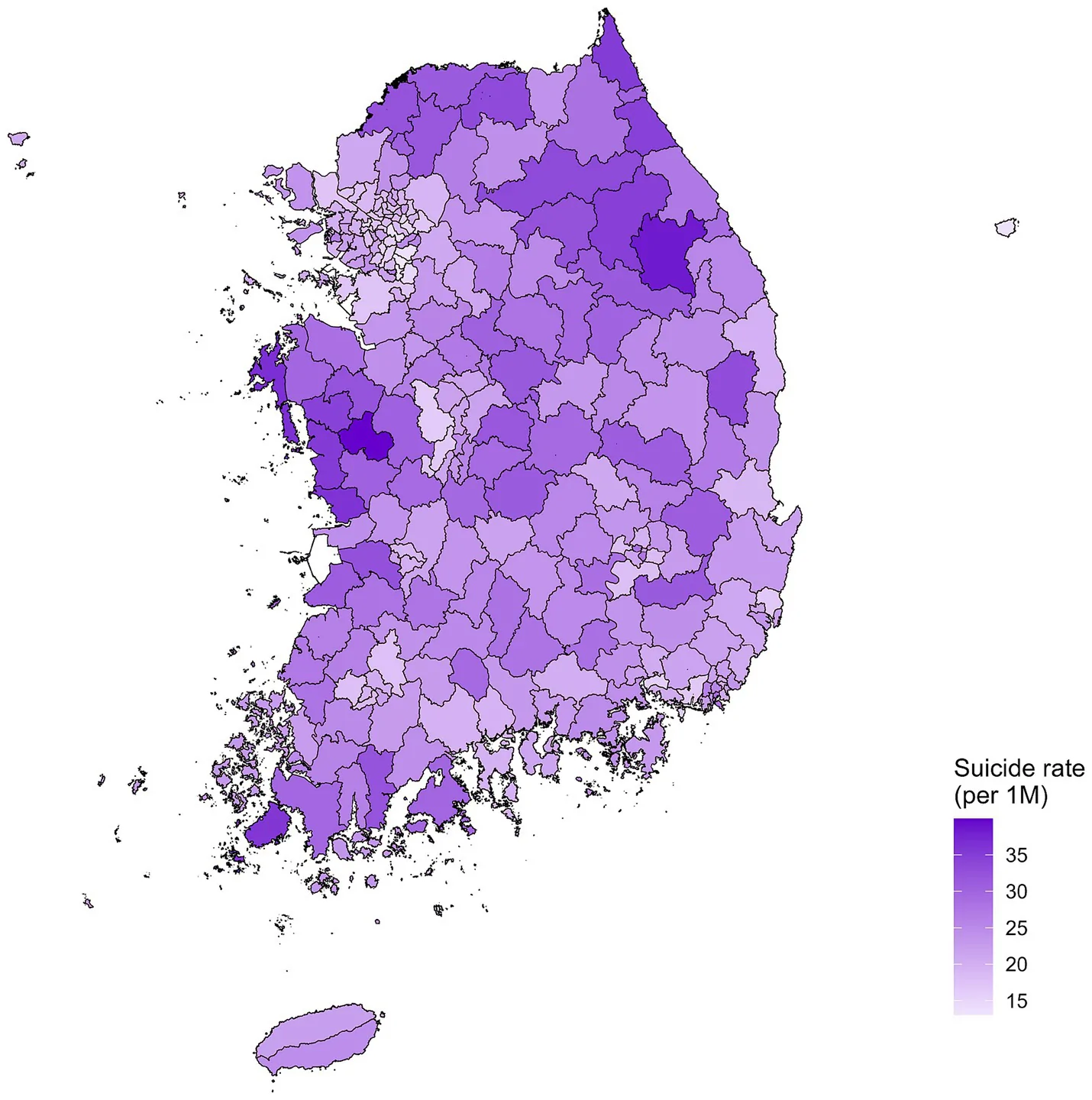
Average suicide rate in South Korea (2015–2019). This figure presents the district-level average suicide rates in South Korea between 2015 and 2019. The suicide rate is defined as the number of suicide deaths per 1 million people.

#### 3.1.2 Thermal inversion

Thermal inversion data were retrieved from ERA5, the fifth-generation European Center for Medium-Range Weather Forecasts reanalysis of global climate and weather over the past eight decades, released by the Copernicus Climate Change Service Climate Data Store.^3^ ERA5 provides gridded atmospheric data at 0.25 × 0.25 degrees and reports air temperature at 37 pressure levels for each hour beginning in 1940. From the 37 pressure levels, air temperatures at the 1,000 hPa (0–100 m) and 975 hPa (300–400 m) levels were used to construct two air-temperature layers at six-hour intervals in each district. Following prior studies (3, 9, 10), a thermal inversion was identified when the temperature at 975 hPa was greater than or equal to that at 1000 hPa.^4^In South Korea, complex topographical and meteorological features—such as mountainous terrain and proximity to surrounding seas—frequently produce atmospheric stagnation phenomena, notably thermal inversion (36, 37). Thermal inversion inhibits convection and limits pollutant dispersion, thereby elevating PM_2.5_ concentrations through physical processes.

This study utilized temperature data from two atmospheric layers, derived from the ERA5 reanalysis grids (0.25° × 0.25°) that were spatially aggregated to the administrative district level. Thermal inversion was defined according to the temperature differential between the two layers at the district level. In addition, thermal inversion was observed at six-hour intervals, yielding four observations per day. The monthly-inversion variable is the count of six-hour intervals in which an inversion was recorded. Thus, this variable reflects the frequency of inversion observations rather than the number of distinct episodes. These observations were aggregated at the district level, and monthly counts of inversion events were compiled to construct the thermal inversion variable. I aggregated the number of thermal inversions at the district-by-month level and matched districts on mortality and air pollution data by district and year.

#### 3.1.3 Air pollution

Concentration data on PM_2.5_ and other air pollutants were obtained from the Korea Environment Corporation’s (K-eco) Air Korea database. The K-eco data include real-time hourly data on PM_2.5_, PM_10_, sulfur dioxide (SO_2_), carbon monoxide (CO), nitrogen dioxide (NO_2_), and ozone (O_3_), as measured by the K-eco pollution monitoring station.^5^ The extensive national coverage of monitoring stations helps reduce measurement errors. District-daily pollution data were constructed by averaging the concentrations measured at all stations within each district in South Korea and by calculating the monthly average pollution concentration for each district, to match the temporal and spatial resolutions of the pollution with the mortality data.

Figure 4 plots the monthly average PM_2.5_ levels in South Korea for 2015–2019. The plot shows substantial temporal variation, with pronounced seasonal fluctuations. A few high-PM_2.5_ episodes are observed, with concentrations close to 40 μg/m^3^ in certain months. These patterns align with meteorological conditions that affect atmospheric dispersion, including thermal inversions and seasonal stagnation. The considerable variation in PM_2.5_ concentrations over time provides meaningful variation for identification in the empirical analysis.

**Figure 4:**
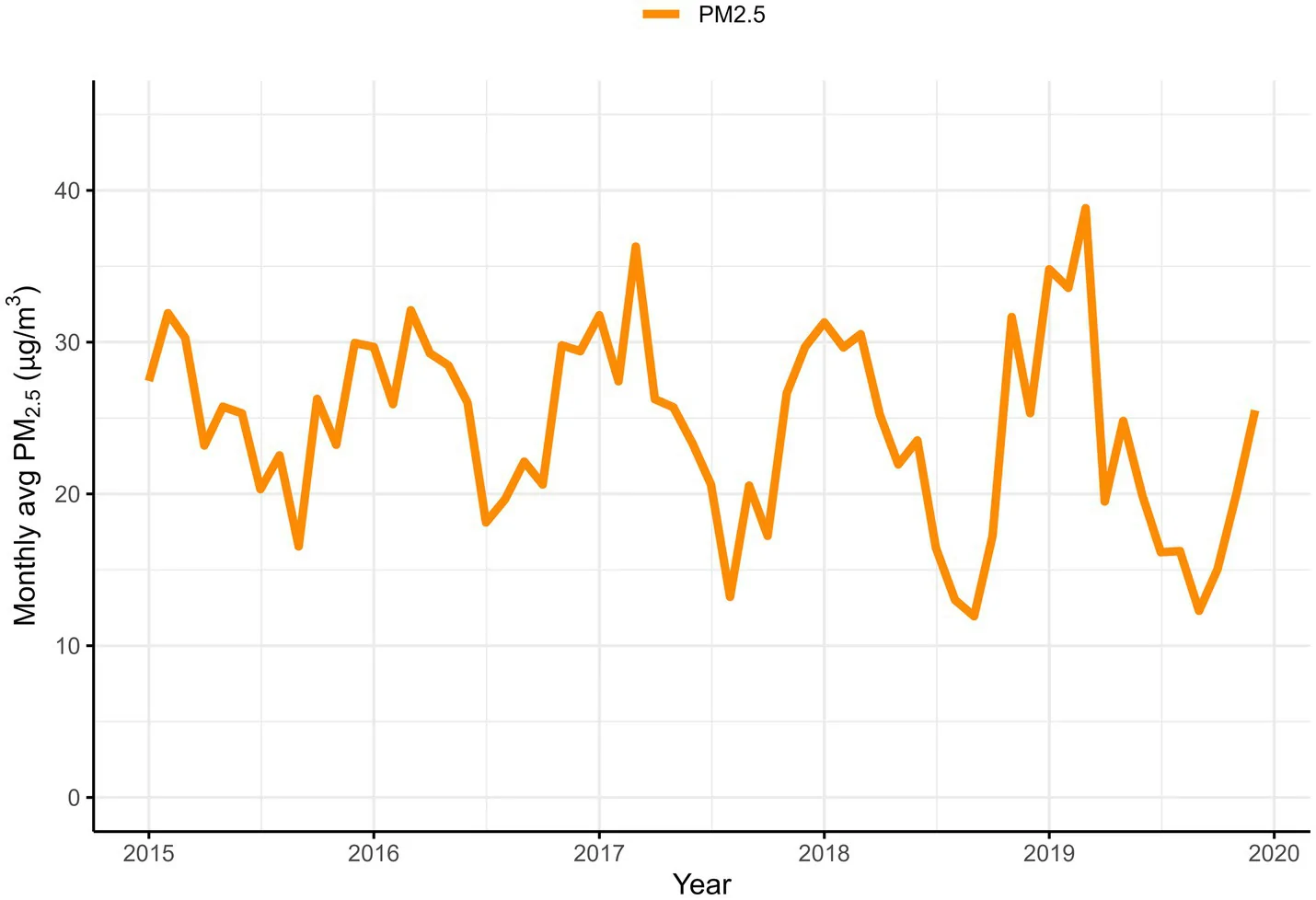
Time trend of PM_2.5_ in South Korea (2015–2019). This figure plots the monthly country-average PM_2.5_ concentration in South Korea from 2015 to 2019, using monthly averages constructed from daily mean PM_2.5_ observations.

#### 3.1.4 Weather conditions

This study utilized weather data provided by the Korea Meteorological Administration (KMA), which operates a network of nationwide weather monitoring stations that record hourly temperature, precipitation, wind speed, and wind direction. Daily average weather values were calculated for each station before constructing the district daily data. Monthly data were calculated at the district level based on the daily average data for analysis. Daily temperatures were categorized into bins to address the nonlinear effects of temperature on suicide mortality^6^ (2024). Similarly, wind direction was grouped into four categories based on South Korea’s geographical location and seasonal wind patterns.

Table 1 presents the descriptive statistics for suicide, air pollution, thermal inversion, and weather conditions. The units of observation are the district and monthly levels from 2015 to 2019 in South Korea. The analytic sample comprises 9.586 district-month observations across 239 districts. This reflects two exclusions from the 14,820 theoretically possible observations: 5,148 observations lacking PM_2.5_ readings due to measurement-related technical issues, and 86 observations missing wind-speed data. The mean monthly suicide mortality rate during this period was 22.52 per 1 million population. The mean monthly average of PM_2.5_ concentration in South Korea is 24.05 μg/m^3^, and the average number of thermal inversions is 26.19, with a standard deviation of 9.09, indicating substantial variation across months.

**Table 1:** Descriptive statistics (2015–2019).

Variable	Unit	Mean	SD	Obs.
Suicide				
Suicide mortality rate	Per million population	22.52	14.78	9,586
Air pollution				
PM_2.5_ concentration	μg/m^3^	24.05	8.25	9,586
Thermal inversions				
Inversions	Number	26.19	9.09	9,586
Weather conditions				
Temperature categories	Days			
Extremely cold		0.10	0.68	9,586
Very cold		0.62	1.86	9,586
Cold		2.32	4.57	9,586
Mild		8.36	9.85	9,586
Warm		15.23	12.15	9,586
Hot		3.44	7.23	9,586
Extremely hot		0.35	1.66	9,586
Precipitation	mm	94.05	94.77	9,586
Wind speed	m/s	1.75	0.69	9,586
Wind direction	Days			
North		13.78	8.02	9,586
East		4.46	4.58	9,586
South		2.77	3.79	9,586
West		9.38	6.39	9,586

The unit of observation is district-month in 2015–2019. The sample (*N* = 9,586) excludes observation with missing PM_2.5_ and wind speed data, as detailed in the text. PM_2.5_ is measured as the monthly average of daily mean concentrations. Precipitation is calculated as the district-daily values were correctly averaged across monitoring stations.

### 3.2 Empirical strategy

This study employed a quasi-experimental design to estimate the effect of air pollution on suicide mortality because conventional OLS estimates can be biased and inconsistent due to measurement error and unobserved confounding. First, PM_2.5_ exposure levels are determined from monitoring station data, which may not accurately reflect individuals’ true exposure (38, 39). Variations in mobility patterns and indoor–outdoor activity can generate discrepancies between measured and actual exposure, with such measurement error tending to attenuate estimated coefficients.

Second, the association between PM_2.5_ and suicide mortality may be confounded by unobserved factors. Although PM_2.5_ may be considered a causal determinant of mental health, this does not ensure the exogeneity of observed exposure values. In practice, air pollution levels are not randomly assigned. For instance, temporal covariates, such as regional economic cycles or employment conditions, may directly affect suicide mortality while simultaneously shaping pollution levels via industrial activity and energy use (6, 40). In such cases, the association between PM_2.5_ and suicide mortality is jointly determined by shared factors, resulting in omitted variable bias or unobserved confounding. Seasonal variation, illustrated in Figures 1, 4, exemplifies this type of confounding (41). In the cold season, PM_2.5_ concentrations and thermal inversions rise, while suicide mortality tends to fall. When seasonal factors jointly influence both variables, simple regression estimates may be biased downward.

Third, behavioral responses to pollution represent another key source of endogeneity. When pollution levels increase, individuals may curtail outdoor activities or modify labor supply as avoidance strategies, and these behavioral adjustments can influence both actual exposure and mental health, thereby biasing simple regression estimates (42, 43).

Thus, although PM_2.5_ is plausibly associated with suicide, observed exposure values may lack exogeneity, thereby undermining the consistency of ordinary least squares (OLS) estimates. By contrast, thermal inversions—physical phenomena resulting from temperature differentials between atmospheric layers—are structurally distinct from PM_2.5_, which is measured as a continuous pollutant concentration. This distinction indicates that thermal inversion is not a proxy for exposure but rather serves as a suitable instrumental variable that offers exogenous variation.

Accordingly, this study uses thermal inversion as an instrumental variable to isolate exogenous variation in PM_2.5_ and to estimate the effect of inversion-driven air pollution suicide mortality (9–12). To address biased estimates, this study uses thermal inversion as an instrumental variable. Thermal inversion limits atmospheric mixing and prevents pollutant dispersion, thereby increasing particulate matter concentrations through a well-established physical process. Accordingly, it meets the relevance condition, which requires that an instrument be strongly correlated with the endogenous variable—in this case, air pollution levels.

The validity of the instrumental variable approach depends not only on relevance but also on the exclusion restriction, which requires independence from unobserved confounders and the absence of any direct effect on the outcome variable (44). In this study, thermal inversion is used to influence PM_2.5_ concentrations by limiting atmospheric mixing; it has been widely employed as an instrumental variable for the same reason in prior studies to identify the health impacts of air pollution (3, 45).

Nevertheless, meteorological factors, such as temperature, may simultaneously influence thermal inversion, air pollution, and suicide mortality, thereby threatening the instrument’s exogeneity. To mitigate this concern, the study controls for key meteorological variables—including temperature, precipitation, and wind speed—and introduces regional and temporal fixed effects to capture unobserved heterogeneity and common shocks. This specification aims to minimize the possibility of thermal inversion affecting suicide mortality through channels other than air pollution. It should be emphasized, however, that the instrumental variable approach rests on identification assumptions that cannot be fully validated with observational data. Consequently, the findings of this study should be interpreted within the bounds of these assumptions. Moreover, because the data are clustered at the provincial level with 17 clusters, the first-stage F-statistic warrants additional caution; the preferred specification’s value (23.9) exceeds conventional thresholds, but it should be interpreted as evidence of relevance rather than a guarantee of instrument validity. Two-stage least squares analysis was conducted to assess the relationship between air pollution and suicide mortality. PM_2.5_ data were compiled by aggregating readings from all air quality monitoring stations within each administrative district. District-level daily averages of PM_2.5_ concentrations were calculated and subsequently aggregated into monthly data. Suicide mortality data were also compiled at the district level and merged with the PM_2.5_ dataset to maintain spatial consistency in the analysis. The thermal inversion variable was constructed from ERA5 reanalysis-based temperature data for atmospheric layers within 0.25° × 0.25° grids. These data were aggregated to the district level, aligning with the spatial unit of the suicide mortality dataset and enabling direct assignment without spatial inconsistency. The first-stage regression linking thermal inversions to the local PM_2.5_ level is as follows:

$$\;P_{\mathrm{it}}=\lambda \;TI_{\mathrm{it}}+f(W_{\mathrm{it}})+\gamma_{i}+\omega_{\mathrm{ps}}+\delta_{y}+\varepsilon_{\mathrm{it}}\;\;\;$$ (1)

Equation 1 measures the air pollution concentration at each district level using the total number of thermal inversions. The dependent variable here is $\;P_{\mathrm{it}}$ , representing the average air pollution level in the district i for month t . The variable of interest is the total number of thermal inversions in a month and district, denoted as $TI_{\mathrm{it}}$ . Thermal inversions are defined using air temperature in the first layer (0–100 m) minus the second layer (300–400 m), with a negative difference indicating thermal inversion during each six-hour period. The number of thermal inversions per month is aggregated at the district level. Coefficient λ measures the association between thermal inversion and air pollution and is crucial in evaluating the relevance of IVs. The equation includes weather variables ( $W_{\mathrm{it}}$ ) such as the number of days by temperature and wind direction bins, second-order polynomials for cumulative precipitation, and average wind speed.^7^ To account for the direct effect of temperature on suicide mortality, the model incorporated the number of days in each temperature interval. This adjustment reflects that thermal inversion primarily occurs at low temperatures. As high temperatures may also be linked to suicide risk, this specification mitigates the confounding effects attributable to temperature. These weather conditions are correlated with thermal inversions and can be linked to suicide (9, 46).

The specification also includes the district ( $\gamma_{i}$ ), province-by-season ( $\omega_{\mathrm{ps}}$ ), and year ( $\delta_{y}$ ) fixed effects. District fixed effects control for district time-invariant characteristics, whereas province-by-season fixed effects control for province attributes across seasons, such as regional heating patterns in winter, seasonal industry operations, or local policy interventions (11). Thermal inversion may be correlated with psychological and behavioral factors, such as diminished sunlight exposure or seasonal affective disorder, which could independently affect suicide risk (47, 48). Sunshine-hour records would provide more direct control for this channel; however, station-level sunshine-hour data in South Korea are substantially sparser than our other weather variables, and incorporating them as a covariate would require dropping a nontrivial share of the analytic sample. The same data limitation applies to other potentially relevant meteorological factors: humidity records are available at only a limited subset of stations and contain substantial missing values, and district-level measures of atmospheric pressure and stagnation suitable for this period were not available. Instead, the study includes seasonal indicators to account for common seasonal fluctuations—including systematic variation in day length—thereby reducing, though not fully eliminating, the likelihood that thermal inversion influences suicide mortality through channels unrelated to air pollution. This study treats this as a reasonable approach given data limitations and refers it as a limitation of the current specification (see Section 5).

Finally, year fixed effects control for time-varying shocks, such as those induced by social and economic changes or policy interventions during the sample period. All standard errors are clustered at the provincial level, corresponding to South Korea’s 17 first-tier administrative divisions (si/do), and all estimates are weighted by district-level population. Although the dependent variable is already a population-normalized rate, weighting remains appropriate because suicide rates in small-population districts are based on few underlying events and are therefore volatile; weighting reduces this noise and targets the effect for the average individual rather than the average district. Suicide mortality is then regressed on the predicted value of local air pollution using Equation 2:

$$Y_{\mathrm{it}}=\beta \hat{P_{\mathrm{it}}}+f(W_{\mathrm{it}})+\gamma_{i}+\omega_{\mathrm{ps}}+\delta_{y}+\varepsilon_{\mathrm{it}}\;\;$$ (2)

Where $Y_{\mathrm{it}}$ is the suicide rate, namely the number of suicides per million population in district *i* in month *t*. The parameter of interest (*β*) represents the estimates of the impact of air pollution on suicide mortality. It estimates the local average treatment effect, which is the average effect of air pollution on suicide for individuals whose changes in the number of thermal inversions within the district alter local pollution exposure (49). After controlling for all variables, thermal inversion must be uncorrelated with other factors beyond pollution levels that also affect suicide rates. Although this restriction could not be verified, weather conditions ( $W_{\mathrm{it}}$ ) are included to control for potential additional confounding factors. Weather variables are included as control variables in Equation 1 because weather can directly affect mental health and suicide rates (46). For example, months with a high frequency of extremely hot days can influence suicide rates by increasing psychological stress or interfering with the activities of daily living. In addition, specific weather patterns can be correlated with the occurrence of both thermal inversions and suicide-related behaviors, which can lead to bias in the estimates if left uncontrolled for. Weather conditions are included in the second-stage regression to account for this, following the same format as defined in Equation 1. The same fixed effects are defined in Equation 1, whereas clustering is conducted at the province level (17 clusters) in Equation 2, and estimates are weighted by district population.

## 4 Results

### 4.1 First-stage results

Table 2 reports the first-stage estimates of the effect of thermal inversions on PM_2.5_ pollution. All regressions control for district and time fixed effects and weather conditions, including days depending on temperature and wind direction bins, second-order polynomials in accumulative precipitation, and wind speed. The specification in column (1) includes district fixed effects for controlling for district-level time-invariant characteristics. Column (2) adds province-by-season fixed effects to control for regional seasonality, reflecting a seasonal pattern of the inversions. In column (3), the specification includes year fixed effects to control for national-level shocks.

**Table 2:** Effect of thermal inversions on air pollution (first stage).

Variable/Statistic	Dependent variable: PM2.5 (μg/m³)		
	(1)	(2)	(3)
Thermal inversions	0.1074^***^	0.0405^***^	0.0446^***^
	(0.0110)	(0.0085)	(0.0091)
Effect relative to the mean (%)	0.447	0.168	0.186
Wald *F*-Statistics	94.7	22.5	23.9
Observations	9,586	9,586	9,586
Controls			
Weather variables	Yes	Yes	Yes
District FE	Yes	Yes	Yes
Province-by-season FE	No	Yes	Yes
Year FE	No	No	Yes

The table regresses district-level pollution measures on thermal inversions during 2015–2019. All regressions include weather, temporal, and district fixed effects. Standard errors are clustered at the provincial level. Significance levels are ^***^ for 1%, ^**^ for 5%, and ^*^ for 10%.

All estimates suggest that thermal inversions have a positive effect on PM_2.5_ levels. The preferred specification in column (3) suggests that one additional thermal inversion in a month increases the PM_2.5_ concentration by 0.0446 μg/m^3^ (95% CI: 0.0267–0.0625) or 0.186%, calculated at the mean. A one standard deviation increase in thermal inversion (9.1 units) increased the concentration of PM_2.5_ by 1.69% relative to the mean. Table 2 reports the Wald *F*-statistic to test for weak instruments (50). The values exceed the commonly cited threshold of 10, suggesting that thermal inversion is a stronginstrument for PM_2.5_ concentrations.

To assess whether the instrument affects PM_2.5_ or broadly influences ambient pollution, this study estimated separate first-stage regressions with PM_10_, SO_2_, CO, NO_2_, and O_3_ as dependent variables, using the same specification as column (3) of Table 2. As reported in Table 3, thermal inversions are positively associated with higher PM_10_, SO_2_, and O_3_ concentrations, consistent with the accumulation of particulate matter and less-reactive gases under a stable inversion layer. In contrast, thermal inversions show no significant relationship with CO (*F* = 0.003) or NO_2_ (*F* = 0.5), pollutants more closely tied to fresh vehicular combustion. This pattern indicates that the instrument does not mechanically trap all ambient pollutants in an undifferentiated manner, though it does affect a broader set of pollutants than PM_2.5_ alone—a point revisited in the Discussion (Section 5). The first-stage relationship is weaker for PM_10_ than for SO_2_ or O_3_, so mixture interpretation does not rest on a uniformly strong first stage for every co-pollutant. It is supported by the strong and significant responses of SO_2_ and O_3_, in addition to PM_2.5_, indicating that the broader conclusion does not depend on the PM_10_ relationship alone.

**Table 3:** Effect of thermal inversions on other pollutants (first stage).

Variable/Statistic	Dependent variable				
	PM_10_ (μg/m^3^)	SO_2_ (ppm)	CO (ppm)	NO_2_ (ppm)	O_3_ (ppm)
Thermal inversions	0.0808^***^	0.00001^***^	0.00001	0.00001	0.00007^***^
	(0.0286)	(0.000001)	(0.00025)	(0.000014)	(0.00001)
Effect relative to the mean (%)	0.187	0.245	0.003	0.048	0.229
Wald *F*-Statistics	8.0	55.6	0.003	0.5	42.2
Observations	9,586	9,586	9,586	9,586	9,586
Controls					
Weather variables	Yes	Yes	Yes	Yes	Yes
District FE	Yes	Yes	Yes	Yes	Yes
Province-by-season FE	Yes	Yes	Yes	Yes	Yes
Year FE	Yes	Yes	Yes	Yes	Yes

The table regresses district-level pollution measures on thermal inversions during 2015–2019. All regressions include weather, temporal, and district fixed effects. Standard errors are clustered at the provincial level. Significance levels are ^***^ for 1%, ^**^ for 5%, and ^*^ for 10%.

### 4.2 Second-stage results

#### 4.2.1 Effect of PM_2.5_ on suicide mortality

Table 4 shows the estimates of the effect of PM_2.5_ pollution on suicide mortality across different specifications. The regression models were estimated using Equation 2. Columns (1) and (2) present the OLS estimates of the relationship between monthly PM_2.5_ pollution and suicide mortality rates for different fixed effects. From the preferred specification in column (2), each 1 μg/m^3^ increase in monthly PM_2.5_ exposure is associated with 0.0315 suicide deaths avoided per million population(95% CI, −0.0549–-0.0081) or a 0.14% decrease relative to the monthly average suicide mortality rate.

**Table 4:** Effect of PM_2.5_ on suicide mortality (second stage).

Variable/Statistic	Dependent variable: suicide mortality			
	OLS Analysis		IV Analysis	
	(1)	(2)	(3)	(4)
PM_2.5_ (μg/m^3^)	−0.0423^***^	−0.0315^***^	0.9763^***^	0.6628^**^
	(0.0155)	(0.0119)	(0.2969)	(0.2847)
Effect relative to the mean (%)	−0.188	−0.140	4.336	2.944
Wald *F*-Statistics	-	-	22.5	23.9
Observations	9,586	9,586	9,586	9,586
Controls				
Weather variables	Yes	Yes	Yes	Yes
District FE	Yes	Yes	Yes	Yes
Province-by-season FE	Yes	Yes	Yes	Yes
Year FE	No	Yes	No	Yes

This table reports the OLS and IV estimates of Equation 2. The dependent variable in all columns is suicide rate, whereas the dependent variable is the number of suicide deaths per million population. Weather controls include the number of days based on the temperature and wind direction bins and second-order polynomials for cumulative precipitation and wind speed. The regression models are weighted using population size. Standard errors clustered by province are reported between parentheses. Significance levels are indicated as *** for 1%, ** for 5%, and * for 10%.

Columns (3) and (4) report the IV estimates of the impact of PM_2.5_ pollution on suicide mortality, suggesting that a 1 μg/m^3^ increase in monthly PM_2.5_ exposure is associated with 0.6628 additional deaths by suicide in a month per million population (95% CI: 0.1049–1.2208) or 2.94% compared to the average suicide mortality rate. Based on the IV estimate (0.6628) in Table 4, column (4), a one-standard-deviation increase in monthly PM_2.5_ pollution (8.25 μg/m^3^) is associated with 5.465 additional suicides per 1 million people. The *F*-statistics in columns (3) and (4) confirm the strength of the first stage, mitigating concerns about weak instrument bias.

The divergence between the OLS and IV estimates likely reflects two sources of bias in the OLS estimate. First, seasonal confounding: PM_2.5_ concentrations peak in the cold season, when suicide mortality is lowest, a pattern consistent with well-documented seasonal cycles in suicide risk unrelated to pollution (41, 46). Second, reverse causality through economic activity: periods of reduced economic activity are associated with both lower pollution and higher suicide risk (40), which would bias the OLS coefficient toward zero or a negative value. The IV approach isolates variation in PM_2.5_ driven specifically by thermal inversions, which is plausibly independent of these confounders, and recovers a positive, significant effect consistent with similar OLS-IV divergences reported in the broader air-pollution-and-suicide literature (1, 6). The main results indicate a positive association between PM_2.5_ and suicide mortality, and the relevance of thermal inversions to PM_2.5_ remains strong (see Appendix Tables A1, A2). The main estimate is also robust to the functional form of the outcome. Re-estimating column (4) with a log(1 + rate) transformation, which accommodates zero values, yields a positive and statistically significant effect consistent with the baseline (Appendix Table A4).

#### 4.2.2 Effect of PM_2.5_ on suicide mortality by gender and age group

This study has found a positive association between PM_2.5_ pollution and suicide mortality in the entire South Korean population. Prior studies illustrate that the impacts of PM_2.5_ on suicide are heterogeneous across demographic characteristics, given that residential segregation and economic and physiological factors increase the risk of suicide for specific groups (6). This study also explored the effect of PM_2.5_ on suicide rates in different demographic groups. Table 5 reports the estimated effect of inversion-driven changes in PM_2.5_ on suicide mortality by gender and age group. Columns (1) and (2) present the differential effect of PM_2.5_ on suicide rates by gender, with the IV estimate in column (1) implying that each 1 μg/m^3^ increase in monthly PM_2.5_ exposure is associated with a significant increase in male suicides of 0.9549 per 1 million male population(95% CI: 0.1472–1.7627). By contrast, the IV estimate for female suicide in column (2) indicates a positive sign but is not statistically significant. This finding is consistent with previous findings reporting a significant association between air pollution and suicide risk among males (14, 51–53).

**Table 5:** Effect of PM_2.5_ on suicide mortality by gender and age group (second stage).

Variable/Statistic	Gender		Age		
	Male	Female	Age 0–14	Age 15–64	Age 65+
	(1)	(2)	(3)	(4)	(5)
PM_2.5_ (μg/m^3^)	0.9549^**^	0.3668	0.2096^**^	0.4849	2.5685^*^
	(0.4121)	(0.2727)	(0.0996)	(0.4708)	(1.3815)
PM_2.5_ Effect (1-SD Increase)	7.873	3.024	1.728	3.998	21.178
Wald *F*-Statistics	23.9	23.9	23.9	23.9	23.9
Observations	9,586	9,586	9,586	9,586	9,586
Controls					
Weather variables	Yes	Yes	Yes	Yes	Yes
District FE	Yes	Yes	Yes	Yes	Yes
Province-by-season FE	Yes	Yes	Yes	Yes	Yes
Year FE	Yes	Yes	Yes	Yes	Yes

This table reports the IV estimates for Equation 2. The dependent variable in all columns is the suicide rate and the dependent variable is the number of suicide deaths per million people. Weather controls include the number of days based on the temperature and wind direction bins and second-order polynomials for cumulative precipitation and wind speed. The regression models are weighted using population size. Standard errors clustered by province are reported between parentheses. Significance levels are indicated as *** for 1%, ** for 5%, and * for 10%.

Table 5 also presents the relationship between air pollution and the three age group categories in columns (3)–(5), namely, those aged 0–14, 15–64, and 65 years and older, respectively. Statistically significant associations are observed for the children (aged 0–14) group in column (3) and the older-adult (aged 65+) group in column (5). For the older adult group, a 1 μg/m^3^ increase in PM_2.5_ exposure is associated with 2.5685 additional suicide deaths per million (95% CI: −0.1392–5.2763). Although this estimate is statistically significant at the 10% level, its 95% confidence interval includes zero, indicating that the data are also compatible with no effect. For the children (aged 0–14) group, a 1 μg/m^3^ increase in PM_2.5_ exposure is associated with 0.2096 additional suicide deaths per million (95% CI, 0.0145–0.4047), which is statistically significant. This estimate should nonetheless be interpreted with caution: suicide among children is a rare event, and the outcome is zero in 98.4% of district-month observations (9,435 of 9,586). With so few non-zero observations, the linear IV estimate for this group is sensitive to a small number of cases and may not be well suited to such a sparse count outcome. The direction and magnitude of these findings are broadly consistent with previous studies documenting associations between air pollution and suicide risk among older adults (18, 54).

#### 4.2.3 Effect of PM_2.5_ on suicide mortality by education and metropolitan area

It is interesting to consider the heterogeneous effects of education level and living in a metropolitan area when estimating the impact of PM_2.5_ on suicide rates. The education level may be a determinant of working conditions, as individuals who work outdoors face increased exposure to air pollution. In addition, people living in the non-metropolitan areas of South Korea face limited job opportunities, social isolation due to demographic aging, and lack of access to medical support systems, all of which can deteriorate their mental health, thereby increasing the risk of suicide (19, 30).

Table 6 presents the IV estimates of the impact of PM_2.5_ on suicide mortality, disaggregated by education level and metropolitan area status. The IV estimate in column (1) suggests that a 1 μg/m^3^ increase in monthly PM_2.5_ pollution is associated with an increase of 0.496 suicide deaths per 1 million inhabitants(95% CI: 0.053–0.939) among individuals with high school education or lower. By contrast, the IV estimate for college education level shows a positive but not statistically significant effect. A significant association between PM_2.5_ pollution and suicide risk among those with a high school education or less has also been reported (5, 14).

**Table 6:** Effect of PM_2.5_ on suicide mortality by education and metropolitan (second stage).

Variable/Statistic	Education		Metropolitan area	
	HS or less	College	Non-Metro	Metro
	(1)	(2)	(3)	(4)
PM_2.5_ (μg/m^3^)	0.4962^**^	0.1666	0.6590^**^	2.1764
	(0.2264)	(0.2392)	(0.3215)	(1.5603)
PM_2.5_ Effect (1-SD Increase)	4.091	1.374	5.434	17.945
Wald *F*-Statistics	23.9	23.9	23.8	0.01
Observations	9,586	9,586	7,556	2,030
Controls				
Weather variables	Yes	Yes	Yes	Yes
District FE	Yes	Yes	Yes	Yes
Province-by-season FE	Yes	Yes	Yes	Yes
Year FE	Yes	Yes	Yes	Yes

This table reports the IV estimates for Equation 2. The dependent variable in columns (1)–(2) is the suicide rate and the dependent variable the number of suicide deaths per million inhabitants in columns (3)–(4). Weather controls include the number of days based on the temperature and wind direction bins, second-order polynomials of cumulative precipitation, and wind speed. The regression models are weighted using the population size. Standard errors clustered by the province are reported in parentheses. Significance levels are indicated as ^***^ for 1%, ^**^ for 5%, and ^*^ for 10%.

Columns (3) and (4) show the estimated effect of PM_2.5_ on suicide rates by type of administrative area. Among non-metropolitan areas, the IV estimate in column (3) implies that each 1 μg/m^3^ in monthly PM_2.5_ is positively associated with an increase in the monthly suicide rate by 0.659 deaths per million inhabitants (95% CI: 0.0289–1.2891). The IV estimate in column (4) is positive but not statistically significant, indicating there is no statistically supported evidence of a relationship between PM_2.5_ and suicide rates in metropolitan areas. These results indicate a significant association between air pollution and suicide mortality in non-metropolitan areas, whereas the estimate for metropolitan areas is not statistically significant and should be interpreted with caution given the weak first stage discussed below.

The weak first-stage result in the metropolitan subsample (*F* = 0.01) appears to reflect substantially lower within-district temporal variation in thermal inversions in metropolitan areas relative to non-metropolitan areas, despite metropolitan districts experiencing inversions more frequently on average. This suggests that thermal inversions have essentially no explanatory power for PM_2.5_ within the metropolitan subsample, meaning the instrument fails to induce meaningful variation in the endogenous variable in this subsample. It is consistent with the urban heat island effect, whereby elevated and more persistent urban temperatures reduce the month-to-month variability of natural inversion formation, thereby weakening the instrument’s explanatory power in this subsample. This study reports the metropolitan estimate for completeness but cautions against causal interpretation given this weak-instrument concern. To clarify where the instrument retains explanatory power across subsamples, Appendix Table A3 reports the first-stage estimates for each subsample used in Tables 4, 5.

## 5 Discussion

Understanding the relationship between PM_2.5_ concentration and suicide mortality is important to reducing health threats at both the individual and public levels. This study employed an IV approach to examine the relationship between PM_2.5_ pollution and suicide rates, using thermal inversions that induce exogenous variation in PM_2.5_ levels to isolate the effect of PM_2.5_ on suicide mortality. The results suggest that an increase in monthly PM_2.5_ concentrations is associated with a significant increase in suicide rates.

Heterogeneity analysis revealed that the effect of air pollution on suicide rates differed according to specific factors. Gender- and age-specific analyses showed a significant increase in suicide rates among males, children, and older adults. In further heterogeneity analysis by education level and metropolitan status, the effect of air pollution on suicide rates was more clearly observed among individuals with high school education or lower and those living in non-metropolitan areas.

These results provide additional evidence of the extant findings on the relationship between PM_2.5_ concentration and suicide rates (5, 6). Based on these findings, threats to ambient air pollution must be addressed as a social problem. The literature focusing on South Korea identified this association but with limited spatial coverage and without explicitly incorporating PM_2.5_ (14, 17, 19).

These findings emphasize the importance of integrating air quality improvements into suicide prevention strategies. Because air pollution exacerbates mental health risks alongside its effects on respiratory and cardiovascular diseases, reducing PM_2.5_ pollution could yield substantial public health benefits. Management strategies targeting vulnerable groups are also required. This study may offer insights for suicide prevention policy in South Korea by pointing to air quality as a relevant environmental factor for mental health. However, because the instrument reflects a broader mixture of pollutants, these findings support integrating air quality improvements into a comprehensive prevention strategy rather than establishing that reducing PM_2.5_ alone would lower suicide mortality. The results suggest that cross-ministerial collaboration between the Ministry of Environment and the Ministry of Health and Welfare is essential for designing effective interventions. However, because the findings of this study are based on a single empirical analysis, causal interpretations should not be derived solely from this evidence. Rather, conclusions should be assessed in the context of accumulated evidence from a broader body of prior research.

An additional factor concerns the interpretation of the IV estimate. Thermal inversions generally restrict the vertical dispersion of pollutants at the surface, not PM_2.5_ alone (see Section 2.2). Consistent with this concern, the first-stage estimates (Table 3) show that inversions significantly increase not only PM_2.5_ but also PM_10_, SO_2_, and O_3_, while showing no significant relationship with CO or NO_2_. Because the instrument induces variation in this broader pollution mixture rather than isolating PM_2.5_, the IV coefficient is more precisely interpreted as the effect of inversion-driven ambient pollution on suicide mortality, rather than the effect of PM_2.5_ in isolation. Nonetheless, given that PM_2.5_ is mechanistically the most plausible driver of the physiological and cognitive pathways discussed in Section 2.1, PM_2.5_ likely represents a primary pathway through which inversion-induced pollution may affect suicide mortality, although contributions from co-varying pollutants cannot be ruled out. This study also has several limitations. First, it examines the short-term association between air pollution and suicide behavior using a monthly dataset. Second, although the control variables were limited to major atmospheric factors, potential confounders were addressed through temporal and spatial fixed effects. Relatedly, this study was unable to include sunshine-hour data as a direct control for psychological and behavioral channels potentially correlated with thermal inversion because station-level sunshine records had substantial missingness; instead, it relied on seasonal fixed effects, which may not fully capture this channel. The same constraint applies to other meteorological factors, such as humidity, atmospheric pressure, and stagnation indices, which cannot be directly controlled due to limited station coverage or data unavailability; their direct effects therefore cannot be fully ruled out. Third, the outcome for the youngest age group (0–14) is highly sparse, with no recorded suicides in the large majority of district-months; the estimate for this subgroup should therefore be regarded as exploratory. Thus, future studies should use high-resolution variables, such as a day-level dataset, and include additional controls, such as national or regional suicide prevention policies, social factors, economic shocks, and more complete meteorological data, including humidity, pressure, and sunshine exposure, where available. Despite these limitations, this study provides evidence consistent with an effect of air pollution on suicide rates, highlighting that improving air quality is the key to protecting lives.

## Data Availability

Publicly available datasets were analyzed in this study. This data can be found at: Weather data: The Korea Meteorological Administration (KMA; https://www.weather.go.kr/w/index.do); Thermal inversion data: Copernicus Climate Change Service (C3S) Climate Data Store (CDS; https://cds.climate.copernicus.eu/datasets/reanalysis-era5-pressure-levels?tab=overview); Suicide mortality data: Korean MicroData Integrated Service (MDIS; https://mdis.kostat.go.kr/ofrData/selectOfrDataDetail.do?survId=19&itmDiv=1&nPage=3&itemId=2006&itemNm=보건); and Concentration data on air pollutants: Korea Environment Corporation’s (K-eco) Air Korea database (https://www.airkorea.or.kr/web/last_amb_hour_data?pMENU_NO=123).

## Footnotes

1 PM_2.5_ refers to fine particulate matter in the air, consisting of particles with a diameter of 2.5 micrometers or smaller. PM_2.5_ pollution causes respiratory and cardiovascular diseases.

2 Suicide is defined as intentional self-harm, classified under KCD codes X60 to X84.

3 The data can be downloaded from https://cds.climate.copernicus.eu/datasets/reanalysis-era5-pressure-levels?tab=overview.

4 The 1,000 hPa and 975 hPa pressure levels correspond to about 100 m and 320 m above the surface under standard atmospheric conditions.

5 Real-time air pollution data by monitoring station—including the concentrations of major pollutants—are available from AirKorea (https://www.airkorea.or.kr/web/last_amb_hour_data?pMENU_NO=123).

6 Temperature bins are based on daily average temperature as follows: ≤ − 10 °C (extremely cold), −10 to −5 °C (very cold), −5 to 0 °C (cold), 0 to 10 °C (mild), 10 to 25 °C (warm), 25 to <30 °C (hot), and ≥30 °C (extremely hot).

7 Second-order polynomials for temperature are included to allow for potential non-linear relationships between temperature and the outcome variable for more flexibly capturing both extreme cold and heat effects.
