# Supplementary material for: Fine particulate matter (PM2.5) and suicide in South Korea: an instrumental variable analysis using thermal inversion
Source: Front Public Health. 2026 Sep 14;14:1909365. doi: 10.3389/fpubh.2026.1909365 (PMC13617119; doi:10.3389/fpubh.2026.1909365)
Supplement: Supplementary file 1 [file Supplementary_file_1.docx]

**Fine Particulate Matter (PM_2.5_) and Suicide in South Korea: An Instrumental Variable Analysis Using Thermal Inversion**

**Supplementary Materials(Appendix)**

Table A1: Effect of Thermal Inversions on Air Pollution (Winter–Early Spring)

| Variable/Statistic |  | Dependent variable: PM_2.5_ (µg/m³) | | |
| --- | --- | --- | --- | --- |
|  |  | (1) |  | (2) |
| Thermal inversions |  | 0.1597^***^ |  | 0.1543^***^ |
|  |  | (0.0164) |  | (0.0184) |
| Effect relative to the mean (%) |  | 0.542 |  | 0.524 |
| Wald *F*-Statistics |  | 94.8 |  | 70.3 |
| Observations |  | 3,883 |  | 3,883 |
| Controls: |  |  |  |  |
| Weather Variables |  | Yes |  | Yes |
| District FE |  | Yes |  | Yes |
| Year FE |  | No |  | Yes |

*Notes*: The table regresses district-level pollution measures on thermal inversions during 2015–2019. All regressions include weather, temporal, and district fixed effects. Standard errors are clustered at the provincial level. The sample is restricted to winter–early spring months. Significance levels are ^***^ for 1%, ^**^ for 5%, and ^*^ for 10%.

Table A2: Effect of PM_2.5_ on Suicide Mortality (Winter–Early Spring)

| Variable/Statistic | Dependent variable: Suicide Mortality | | | | |
| --- | --- | --- | --- | --- | --- |
|  | OLS Analysis | |  | IV Analysis | |
|  | (1) | (2) |  | (3) | (4) |
| PM_2.5_ (µg/m³) | -0.0291 | -0.0201 |  | 0.3306^***^ | 0.2648^**^ |
|  | (0.0179) | (0.0145) |  | (0.1184) | (0.1215) |
| Effect relative to the mean (%) | -0.133 | -0.091 |  | 1.504 | 1.205 |
| Wald *F*-Statistics | - | - |  | 94.8 | 70.3 |
| Observations | 3,883 | 3,883 |  | 3,883 | 3,883 |
| Controls: |  |  |  |  |  |
| Weather Variables | Yes | Yes |  | Yes | Yes |
| District FE | Yes | Yes |  | Yes | Yes |
| Year FE | No | Yes |  | No | Yes |

*Notes*: This table reports the OLS and IV estimates of Equation (2). The dependent variable in all columns is suicide rate, whereas the dependent variable is the number of suicide deaths per million population. Weather controls include the number of days based on the temperature and wind direction bins, and second-order polynomials for cumulative precipitation and wind speed. The regression models are weighted using population size. Standard errors clustered by province are reported in parentheses. The sample is restricted to winter–early spring months Significance levels are indicated as *** for 1%, ** for 5%, * for 10%.

Table A3: First-Stage Regressions by Subsample

|  | Dependent variable: PM_2.5_ (µg/m³) | | |
| --- | --- | --- | --- |
| Variable/Statistic | Full Sample  (Table 4 / Table 5-Education) | Non-Metro  (Subsample) | Metro  (Subsample) |
| Thermal inversions | 0.0446^***^ | 0.0472^***^ | 0.0018 |
|  | (0.0091) | (0.0097) | (0.0171) |
| Wald *F*-Statistics | 23.9 | 23.8 | 0.01 |
| Observations | 9,586 | 7,556 | 2,030 |
| Controls: |  |  |  |
| Weather Variables | Yes | Yes | Yes |
| District FE | Yes | Yes | Yes |
| Province-by-season FE | Yes | Yes | Yes |
| Year FE | Yes | Yes | Yes |

*Notes*: The table regresses district-level pollution measures on thermal inversions during 2015–2019. All regressions include weather, temporal, and district fixed effects. Metropolitan/Non-Metropolitan columns use actual subsamples with distinct first-stage regressions. Standard errors are clustered at the provincial level. Significance levels are ^***^ for 1%, ^**^ for 5%, and ^*^ for 10%.

Table A4: Robustness of the IV Estimate to Outcome Transformations

|  | Dependent variable: Suicide Mortality | | |
| --- | --- | --- | --- |
| Variable/Statistic | (1)  Suicide rate (baseline) | (2)  log(1 + rate) | |
| PM_2.5_ (µg/m³) | 0.6628^**^ | 0.0303^**^ | |
|  | (0.2847) | (0.0148) | |
| Wald *F*-Statistics | 23.9 | 23.8 | |
| Observations | 9,586 | 7,556 | |
| Controls: |  |  |  |
| Weather Variables | Yes | Yes | |
| District FE | Yes | Yes | |
| Province-by-season FE | Yes | Yes | |
| Year FE | Yes | Yes | |

*Notes*: Column (1) uses the baseline suicide mortality rate (deaths per million); column (2) applies the log(1 + rate) transformation, which accommodates zero values. Both columns report IV estimates of Equation (2) using thermal inversions as the instrument, with the full set of weather controls and fixed effects. Standard errors are clustered at the provincial level. Significance levels are ^***^ for 1%, ^**^ for 5%, and ^*^ for 10%.
